# Methane Production Characteristics of an Anaerobic Co-Digestion of Pig Manure and Fermented Liquid Feed

**DOI:** 10.3390/molecules27196509

**Published:** 2022-10-02

**Authors:** Farida Hanum, Yoichi Atsuta, Hiroyuki Daimon

**Affiliations:** 1 Department of Applied Chemistry and Life Science, Toyohashi University of Technology, Toyohashi 441-8580, Japan; 2 Department of Chemical Engineering, Faculty of Engineering, Universitas Sumatera Utara, Medan 20155, Indonesia; 3 Research Center for Agrotechnology and Biotechnology, Toyohashi University of Technology, Toyohashi 441-8580, Japan

**Keywords:** anaerobic co-digestion, methane production, co-substrate, pig manure, fermented liquid feed

## Abstract

Methane production characteristics of anaerobic co-digestion of pig manure (PM) and fermented liquid feed (FLF) were investigated in a continuous digester under mesophilic conditions. The experiment followed three phases. PM alone was digested in phase I. In phases II and III, PM and FLF were mixed in a ratio of 95:5 and 90:10 (% *v*/*v*), respectively. The specific methane yields (SMYs) during phases I, II, and III were 238, 278, and 326.8 mLCH_4_·gVS^−1^-added, respectively. It was due to the effect of balancing the feedstock carbon-to-nitrogen ratio by adding FLF. This improvement can also be attributed to the readily biodegradable compounds in the FLF. The higher SMY obtained in this study showed a positive synergistic effect in the anaerobic co-digestion of PM and FLF. The results also indicate that adding the FLF positively affected and maintained a constant pH level, avoiding volatile fatty acid accumulation and ammonia inhibition in the anaerobic digestion (AD). Thus, this study provides valuable information regarding the usage of unused or wasted FLF as a co-substrate for the practical AD of PM. The production of fermented liquid additives such as FLF to improve the methane production from the AD of PM is a potential novel alternative to food waste recycling in Japan, besides compost and animal feeding.

## 1. Introduction

Recently, an increase in food waste has become a global concern. In Japan, around 25.31 million tons of food waste was generated in 2018 from food manufacturing, retail, and consumer households [1]. Appropriate food waste management practices should be implemented to minimize the environmental impacts and maximize social and economic benefits. In Japan, recycling food waste as compost and animal feed is preferred. However, composting food waste still presents several issues, such as relatively low price, high-quality demand by farmers, and a shortage of cropland for application [2,3,4]. Thus, the leftover compost is often discharged rather than used for cropland. Therefore, recycling food waste into fermented liquid feed (FLF) for pigs was considered a possible alternative for many years. To promote FLF production, the Ministry of Agriculture, Forestry and Fisheries (MAFF) issued the feed safety law in August 2006. A method for producing, collecting, transporting, and storing raw materials was established [5]. Although food waste can be collected in relatively large quantities, it is difficult to produce FLF with consistent quality in terms of its nutritional value due to the variation in source availability. Additionally, the government established a new standard of operation act to ensure the safety of FLF and improve its quality in August 2020 [6]. This requires heat treatment of up to 90 °C for 60 min during the production of FLF. This might result in insufficient profit generation for the food waste recycling company due to the need for additional costs. Therefore, the use of unused or waste FLF, including food waste, remains a challenge.

Pig manure (PM) is a plentiful source of organic compounds that can be used as feedstock in anaerobic digestion (AD). It contains several nutrients required for bacterial growth. PM also has a high buffering capacity, which possibly protects AD against failures due to the accumulation of volatile fatty acids (VFAs) [7,8,9]. However, the high ammonia concentration may inhibit bacterial activity in AD [10,11,12,13,14]. Thus, it is preferable to co-digest PM with organic waste containing high carbon to improve the carbon-to-nitrogen (C/N) ratio, dilute the inhibitory effect of ammonia, and enhance the macro and micronutrient balance in the feedstocks [15,16].

A few continuous-scale studies have reported the potential of methane production from the anaerobic co-digestion (AcoD) of PM with various co-substrates, such as food waste and food processing byproducts. Several potential co-substrates have been examined to assess the effect of varying feedstock composition on increasing methane yield and improving the AD process performance. Dennehy et al., 2018 evaluated the effect of varying PM with food waste mixing ratio on methane yield [17]. They discovered that the feedstock composition of 60:40 (volatile solid basis) enhanced methane yield significantly. Molinuevo-Salces et al., 2012 reported that using vegetable processing wastes as co-substrate with a feedstock ratio of 50:50 (dry weight basis) could improve methane yield up to 3-fold [18]. Furthermore, Kaparaju et al., 2005 used potato tuber and its industrial byproduct as co-substrate [19]. They observed that the highest specific methane yield (SMY) was obtained at a feedstock composition of 80:20 (volatile solid basis). However, obtaining suitable co-substrates and mixture ratios is still a major challenge in the AcoD of PM.

Thus, considering the abovementioned factors, FLF seems to be an interesting and unexplored co-substrate for the AD of PM. FLF contains a readily biodegradable organic fraction and a higher C/N ratio than PM. Adding FLF as a co-substrate in the AD of PM is expected to increase the nutrient balance, reduce ammonia inhibition, and enhance methane yield. This study is the first attempt to investigate the characteristics of methane production by AcoD of PM and FLF. The effect of the organic loading rate (OLR) considering the change in substrate mixing ratios of PM to FLF on the AD process performance was investigated in this study using a continuous stirred tank reactor operated at mesophilic temperature (38 °C). Additionally, using unused or wasted FLF as an additive to improve methane production is expected to become a new method for recycling food waste in Japan, besides compost and animal feeding.

## 2. Materials and Methods

### 2.1. Substrates and Inoculum

The primary substrate, PM, comprising feces, urine, and wash water, was obtained from a pig farm in Toyohashi, Japan, where up to 1,000 pigs are bred. FLF, as a co-substrate, was collected from Komasuya Co. Ltd., a food waste company (Nagoya, Japan). Anaerobic sludge as inoculum was procured from two anaerobic reactors, a mesophilic AD plant (300 m^3^ of capacity)-treated PM, and a mesophilic AD plant (15 m^3^ of capacity)-treated grease trap. Thus, it was mixed at 1:1 (% *v*/*v*). The PM, FLF, and inoculum were filtered through a 2 mm mesh to remove coarse particles.

### 2.2. Reactor Operation

This experiment used an MBF-1000 ME reactor for microorganisms (Eyela, Japan) with a working volume of 7 L. The temperature was maintained at 37 ± 1 °C by a water jacket and was operated under mixing conditions (60 rpm) by two stainless steel propellers. The amount of biogas was measured daily using a wet gas meter W-NK-1 (Shinagawa, Japan), and iron oxide was installed through a biogas pipeline system to trap hydrogen sulfide from the produced biogas. The reactor operated in three phases by differences in substrate PM:FLF mixture ratios and OLR. Start-up and phase I were fed only with PM, while phase II was operated with PM:FLF at a ratio of 95:5 (% *v*/*v*) equivalent to 74:26 (volatile solid basis). Finally, phase III was operated with a mixture ratio of 90:10 (% *v*/*v*) equivalent to 59:41 (volatile solid basis). During phase I, the reactor was digested at an OLR of 1.16 gVS·Ld^−1^. While in phases II and III, the reactor was performed at an OLR of 1.36 and 1.58 gVS·Ld^−1^, respectively. During the start-up period, the hydraulic retention time (HRT) was set at 40 days. Thereafter, it decreased gradually to 30 days until the end of the operation period. The reactor was purged with nitrogen gas for about 5 min to remove oxygen before the reactor operation.

### 2.3. Analytical Methods

Total solid (TS), volatile solid (VS), and volatile suspended solid (VSS) were measured according to standard methods (APHA, 2005). Dissolved organic carbon (DOC) concentrations were determined using a TOC-L 500 TOC analyzer (Shimadzu, Japan) under 680 °C combustion temperature. The concentrations of VFAs were measured using a high-performance liquid chromatography (HPLC) prominence with a Shimadzu Shim-Pak SCR-102 H column (Shimadzu, Japan). The column temperature was 40 °C. The eluent composition was 5 mM p-toluenesulfonic acid, 20 mM Bis-Tris acid, and 100 μM EDTA, and the flow rate was set to 0.8 mL·min^−1^. The ammonium ion (NH_4_^+^) concentration was measured using the HPLC Prominence (Shimadzu, Japan) with a Shodex IC YK-421 column (Showadenko, Japan) at 40 °C. The eluent composition was 4 mM phosphoric acid, and the flow rate was set to 0.8 mL·min^−1^. Total carbon and nitrogen were analyzed using an elementary analysensysteme GmbH-Vario EL III CN Analyzer (Langenselbold, Germany) at 950 °C and with an oxygen gas flow rate of 25 mL·min^−1^. The CO_2_ and CH_4_ concentrations in the biogas were measured using a GC-8A gas chromatograph (GC) equipped with a thermal conductivity detector and Shincarbon-ST column (Shimadzu GLC, Japan). Helium was utilized at the carrier gas for the GC at a flow rate of 30 mL·min^−1^. The detector, column oven, and injection temperature were 200 °C, 50 °C, and 200 °C, respectively. All statistical analyses were completed using Microsoft Excel 2016.

## 3. Results and Discussion

### 3.1. Wastes Characterization

Table 1 describes the physicochemical characterizations of PM and FLF used in this study. The pH values of the PM and FLF were 6.3 and 3.5, respectively. The C/N ratio of the PM used was lower than the FLF. PM’s free ammonia and ammonium ion concentrations were 3.6 and 2260 mg·L^−1^, respectively, whereas the free ammonia (NH_3_) concentration of the FLF was not detected. The FLF used as a co-substrate shows little potential for promoting ammonia inhibition during the AD process since acidic materials contain low ammonia concentration. Therefore, the mixture ratio of PM and FLF was investigated under three conditions (Table 2).

### 3.2. pH Values during Anaerobic Digestion

**Figure 1:**
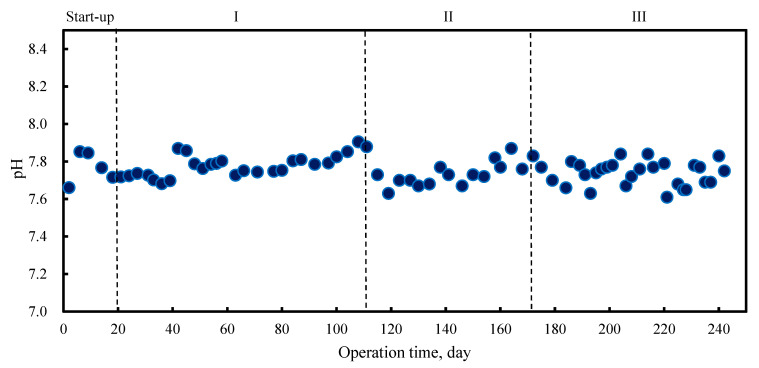
pH values during anaerobic digestion process.

The characteristics of a mesophilic anaerobic reactor on the co-digestion of PM and FLF were investigated at OLRs of 1.17 gVS·Ld^−1^, 1.37 gVS·Ld^−1^, and 1.58 gVS·Ld^−1^, at phase I, II, and III, respectively. Different OLRs were set by changing the mixture ratios of the PM and FLF. The ammonia functions in the PM caused the pH value in the reactor to increase gradually to an alkaline (pH 7.9) (Figure 1). The transfer to the alkali side was slightly suppressed (pH 7.7) through the addition of an acidic substance such as FLF. It has been proven that the optimal range of pH in the AD process is approximately 6.5–8.0 [20,21,22], which is supported by this study. Despite no pH adjustment in this study, the pH values during the entire experiment were relatively stable, around neutral to weak alkali. FLF, as a carbohydrate-rich substrate, could be easily converted into VFAs. However, as a protein-rich substrate, the PM provides the buffering capacity to counter the pH decline in the reactor.

### 3.3. Ammonia Concentrations

**Figure 2:**
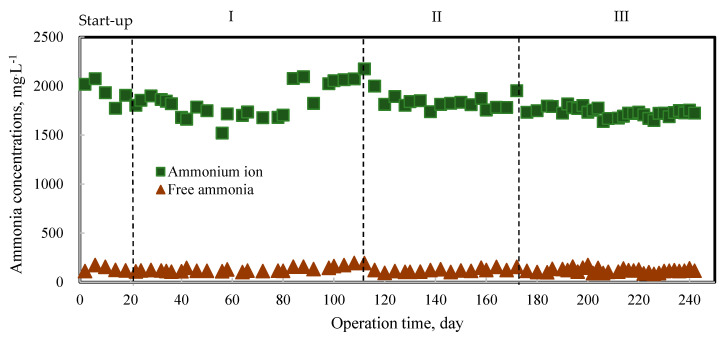
Ammonia concentrations during anaerobic digestion process.

Nitrogen is essential for bacterial growth, while ammonia is an essential source of nitrogen. However, high concentrations of ammonia are toxic for anaerobic bacteria, thus causing inhibition of the AD process. The degradation of amino acids during acidogenic mainly produced total ammonia, consisting of NH_3_ and NH_4_^+^. Compared to NH_4_^+^, NH_3_ has strong inhibitory effects on methanogens [23,24]. A slight accumulation of NH_3_ (maximum concentration: 196 mg·L^−1^) in the reactor was observed toward the end of phase I. It was maintained below 150 mg·L^−1^ in phase III (Figure 2). Previous studies reported that the AD of livestock manure was inhibited by NH_3_ concentrations above 230 mg·L^−1^ [25,26]. Following the NH_3_ concentration, NH_4_^+^ concentration increased to 2200 mg·L^−1^ at the end of phase I and was maintained below 1900 mg·L^−1^ in phase III. A previous study reported that NH_4_^+^ concentration exceeding 2500 mg/L causes inhibition in the AD process [27,28]. The addition of FLF with a higher C/N ratio (20) compared to the PM (5) affected the balance of the C/N ratio of the feedstock, resulting in reduced ammonia concentration below the inhibition threshold. Such effects have also been reported in AcoD of PM with other organic waste, such as vegetable processing waste and potato industrial byproducts [18,19].

### 3.4. Volatile Fatty Acids

**Figure 3:**
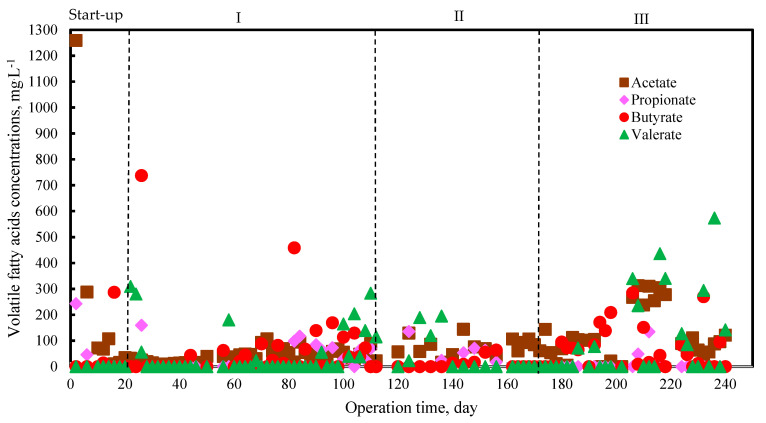
Volatile fatty acids concentrations during anaerobic digestion process.

The concentration of VFAs can be considered reliable for process monitoring in the liquid phase [29,30]. VFAs are the main intermediate products during AD of organic wastes. However, VFAs can be accumulated at high organic loading, resulting in a decrease in pH and leading to digester failure. Therefore, the concentration of VFAs, specifically acetate and propionate, were considered one of the control parameters in the liquid phase. In this study, the major VFAs included acetate, propionate, butyrate, and valerate. VFAs, such as acetate, propionate, and butyrate, temporarily accumulated initially and were gradually consumed during the end of phase I (Figure 3). Acetate, butyrate, and valerate also slightly accumulated during phase III in the range of 300–570 mg·L^−1^. The accumulation occurred because the conversion of VFAs to methane was not completely accomplished under the tested conditions. However, toward the end of phase III, VFAs were present in small amounts, and the concentration of acetate and propionate were observed to be less than 350 and 200 mg·L^−1^, respectively. Previous studies reported that the concentration of acetate and propionate, up to 1500 mg·L^−1^ and 900 mg·L^−1^, significantly inhibited the AD process [31,32]. The low acetate and propionate concentrations might be due to the acclimatization of microbiota to the OLR set from 1.17 to 1.58 gVS·Ld^−1^ in this study. Previous studies reported the optimum OLR of livestock manure and food waste continuously under mesophilic conditions around 1.5–3 gVS·Ld^−1^ [17,33]. However, the OLR can be varied depending on the type of feedstock being treated.

The VFAs monitoring in long-term anaerobic co-digestion of PM and FLF did not indicate an organic overload. Additionally, the stabilization of low VFAs concentration, indicating the stability of the AD process, due to VFAs produced from hydrolysis and acidogenic can be consumed by methanogenic in time.

### 3.5. Methane Production from Anaerobic Co-digestion of Pig Manure and Fermented Liquid Feed

**Figure 4:**
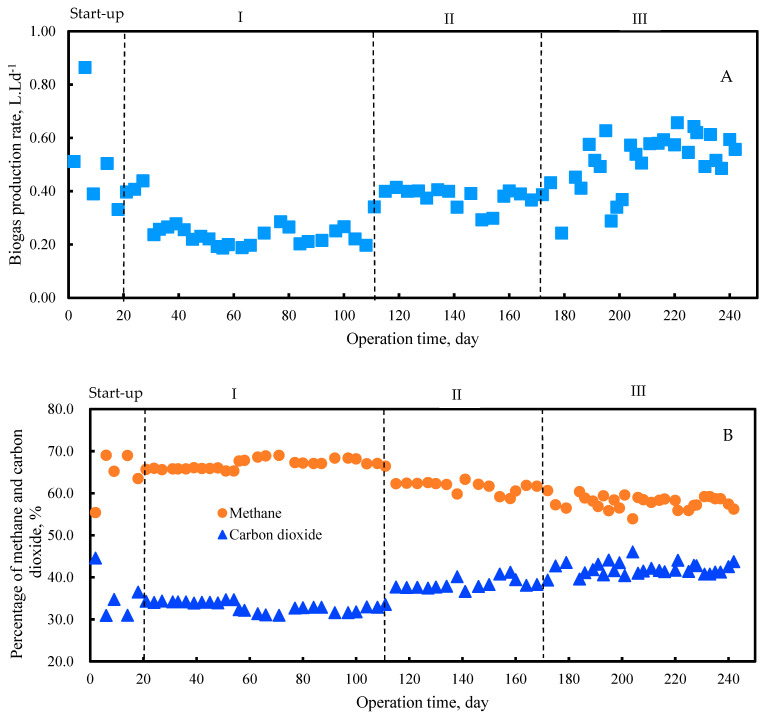
Biogas production rate and percentage of methane and carbon dioxide in the produced biogas (**A**) biogas production rate, (**B**) percentage of methane and carbon dioxide.

The biogas production rate tended to increase with increasing the OLR set in this study (Figure 4A). The biogas production rate of phase I was steadily generated between 0.18 and 0.27 L·Ld^−1^ until the end of phase I, with methane concentrations between 65% and 69%. At the end of phase II, the biogas production rate reached 0.38 L·Ld^−1^ with a methane concentration of 60% (Figure 4A,B). Moreover, the maximum biogas production rate of 0.51 L·Ld^−1^ was obtained during phase III. It showed up to 93% higher mono-digestion of PM in phase I. However, the methane concentration slightly decreased from 66% in phase I to 57% in phase III. This can be attributed to the slight accumulation of VFAs due to the addition of FLF, indicating the slow conversion of VFAs into methane. Moreover, the oxygen content of the FLF was converted into carbon dioxide, increasing the carbon dioxide concentration in the biogas.

**Figure 5:**
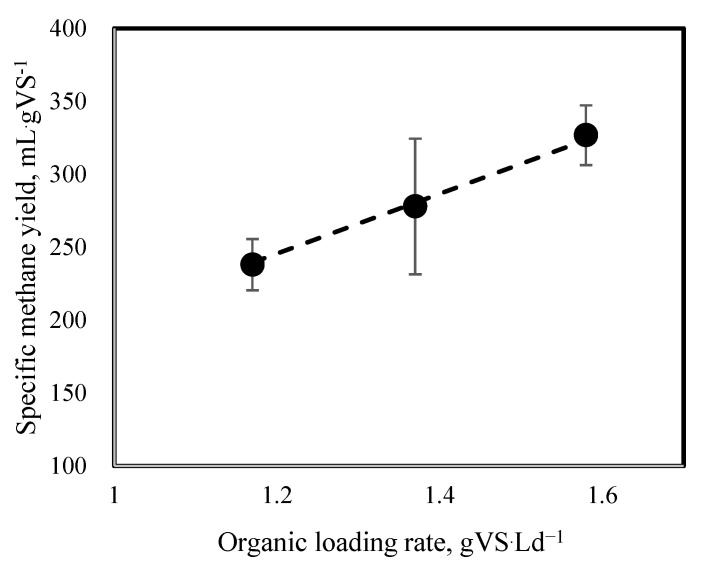
Specific methane yield values at organic loading rate tested in this study.

Thus, concurrent with the production rate, the SMY increased linearly during the experiment. The SMY of phases I, II, and III were observed up to 238, 278, and 326.8 mLCH_4_·gVS^−1^, respectively (Figure 5). By substrate mixture ratio of 90:10 (% *v*/*v*), which is equivalent to 59:41 (VS basis) in phase III, the SMY was enhanced by 37% compared to mono-digestion of PM. Balancing the feedstock C/N ratio by adding FLF resulted in enhanced methanogenic activity. The improvement can also be attributed to a higher readily biodegradable compound in the FLF. Even though the SMY from mono-digestion of the FLF is still unknown, the greater SMY obtained in the co-digestion indicated a positive synergistic effect in the PM and FLF AcoD. However, previous research using a PM to rice straw mixture ratio of 1:1 (VS basis) reported the synergistic effect by increasing the SMY up to 23.7% compared to the mono-digestion of PM and rice straw separately [34]. Xie et al., 2017 also demonstrated a marginal synergistic effect of increasing the SMY by 12% from AD of PM and grass silage mixture ratio of 1:1 (VS basis) compared to the mono-digestion of PM [35]. They reported that the synergistic effect was associated with balancing the feedstock C/N ratio by adding a co-substrate with a high C/N ratio.

Table 3 compares the SMY from AcoD under mesophilic conditions in the continuous operation between this study and several previous reports. The SMY found in this study generally agrees with the SMYs measured in the studies using food waste and industrial potato byproducts as co-substrates [17,19]. Despite the differences in the co-substrate used, the mixture ratio and OLR were almost similar to this study. Other studies reported a lower SMY when using cassava pulp and vegetable processing waste as co-substrate at a low HRT of 15 and 25, respectively [18,36]. Although the OLR set in this study was relatively low, the high content of easily degradable organic matter in FLF resulted in higher SMY.

### 3.6. Volatile Solid Removal

**Figure 6:**
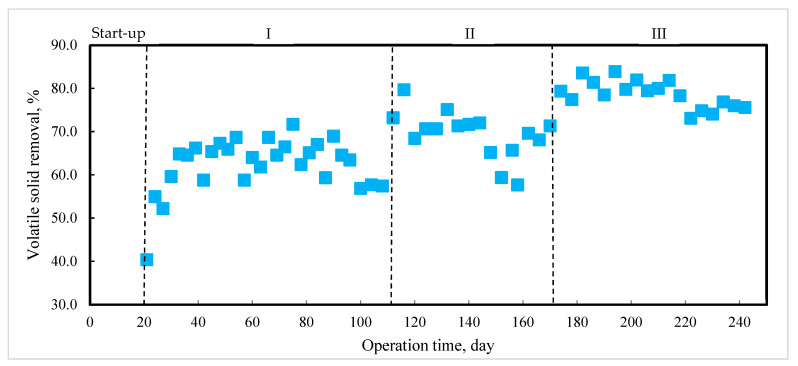
Volatile solid removal during anaerobic digestion process.

Besides enhancing methane production, AD performance could be improved through VS removal. The average of VS removals obtained in phases I, II, and III were 62.3%, 69.3%, and 78.6%, respectively (Figure 6). Panichnumsin et al., 2010 reported that VS removals of about 60% by using PM and cassava pulp operating with an HRT of 15 days and OLR 3.5 gVS·L^−1^·day ^−1^ [36]. Cuetos et al., 2011 obtained the VS removal range of 36%–53% from the AD of PM and energy crops residues operating with HRT of 30 days and OLR 1.2 to 2.3 gVS·L^−1^·day^−1^ [9]. Generally, the VS removal of food waste mono-digestion was approximately 80%. Since the FLF is produced from food waste, the VS removal of the FLF could be similar to or lower than the food waste. Although the VS removal of PM mono-digestion was 62.3% in this study, following the estimation of food waste mono-digestion, the VS removal of PM and FLF co-digestion in phase III was approximately 69.5%. The higher VS removal of around 78.6% obtained in this study indicates an improvement in the PM degradation by adding FLF with a high content of easily degradable organic matter. Thus, higher VS removal provides further evidence of a positive synergistic effect on PM and FLF co-digestion. However, the mechanism of the synergistic effect requires further investigation.

## 4. Conclusions

The PM and FLF feedstock were successfully co-digested using a continuously stirred tank reactor under the test conditions. The AcoD of PM and FLF positively maintained a constant pH level, reducing VFAs accumulation and ammonia concentration. The FLF as a carbohydrate-rich substrate was converted into VFAs, and PM as a protein-rich substrate provided a suitable buffering capacity to prevent the pH decline. The mixture ratio of PM to FLF 90:10 (% *v*/*v*) at an OLR of 1.58 gVS·Ld^−1^ showed higher SMY up to 37% compared with mono-digestion of PM. The balancing C/N ratio of feedstock and higher readily biodegradable compounds in FLF might be the primary cause of improvement in methane production. The higher SMY obtained in this study indicated a positive synergistic effect in the AcoD of PM and FLF. However, the mechanism of the synergistic effect needs to be further investigated. In addition, using FLF as an additive for the AD of PM has the potential to become a new alternative to recycling food waste in Japan, besides compost and animal feeding.

## Figures and Tables

**Table 1:** Characteristics of substrates and inoculum used in this study.

Parameters	Unit	PM	FLF	Inoculum
pH	-	6.3	3.5	7.8
Total solid	%	4.17	14.38	1.77
Volatile solid	%	1.54	13.71	1.17
TN	(mg·L^−1^)	3665	2230	3807
TC	(mg·L^−1^)	15,600	38,592	3705
DOC	(mg·L^−1^)	15,570	38,563	2240
C/N ratio	-	5.2	19.7	6.6
NH_3_	(mg·L^−1^)	3.6	0	94.8
NH_4_^+^	(mg·L^−1^)	2260	130	1955
Acetate	(mg·L^−1^)	4078	2472	220.86
Propionate	(mg·L^−1^)	1880	386	0
Butyrate	(mg·L^−1^)	0	69.6	0
Valerate	(mg·L^−1^)	0	66.2	0

PM: pig manure. FLF: fermented liquid feed; TN: total nitrogen; TC: total carbon; DOC: dissolved organic carbon.

**Table 2:** Mixture ratio of pig manure (PM) and fermented liquid feed (FLF) during anaerobic co-digestion process.

Parameters	Start-Up	Phase I	Phase II	Phase III
Operation period (days)	1–20	21–111	112–172	173–242
Organic loading rate (gVS·Ld^−1^)	0.87–1.17	1.17	1.37	1.58
Mixture ratio of PM to FLF (% *v*/*v*)	100:0	100:0	95:5	90:10
Mixture ratio of PM to FLF (VS basis)	100:0	100:0	74:26	59:41

**Table 3:** Comparison of anaerobic co-digestion results from this study with data reported in the literature.

Study	Kaparaju et al. (2005) [19]	Panichnumsin et al. (2010) [36]	Molinuevo-Salces et al. (2012) [18]	Dennehy et al. (2018) [17]	Present Study	
Substrates	Pig manure: Potato industrial by-product	Pig manure: Cassava pulp	Pig manure: Vegetable processing wastes	Pig manure: Food waste	Pig manure: Fermented liquid feed	
Feedstock mixing ratio	80:20	50:50 (VS basis)	50:50 (dw basis)	60:40 (VS basis)	74:26 (VS basis) 95:5 (% *v*/*v*)	59:41 (VS basis) 90:10 (% *v*/*v*)
OLR (gVS·Ld^−1^)	2	3.5	0.59	1.5	1.16	1.58
HRT (d)	25–26	15	25	29	30	30
SMY (mLCH_4_·gVS^−1^ added)	330	290	285	333	280.4	332
GPR (L·Ld^−1^)	NR	1.67	0.25	NR	0.35	0.56
CH_4_ (%)	62	59	55	NR	60	57

OLR: organic loading rate; HRT: hydraulic retention time; NR: not reported; SMY: specific methane yield; GPR: gas production rate; dw: dry weight.

## Data Availability

Not applicable.

## Sample Availability

Samples are not available from the authors.
